# Revealing Whole-Brain Causality Networks During Guided Visual Searching

**DOI:** 10.3389/fnins.2022.826083

**Published:** 2022-02-18

**Authors:** Christian M. Kiefer, Junji Ito, Ralph Weidner, Frank Boers, N. Jon Shah, Sonja Grün, Jürgen Dammers

**Affiliations:** ^1^Institute of Neuroscience and Medicine (INM-4), Forschungszentrum Jülich GmbH, Jülich, Germany; ^2^Institute of Neuroscience and Medicine (INM-6), Institute for Advanced Simulation (IAS-6), Forschungszentrum Jülich GmbH, Jülich, Germany; ^3^Faculty of Mathematics, Computer Science and Natural Sciences, RWTH Aachen University, Aachen, Germany; ^4^Jülich Aachen Research Alliance (JARA)-Brain – Institute Brain Structure and Function, Institute of Neuroscience and Medicine (INM-10), Forschungszentrum Jülich GmbH, Jülich, Germany; ^5^Institute of Neuroscience and Medicine (INM-3), Forschungszentrum Jülich GmbH, Jülich, Germany; ^6^Institute of Neuroscience and Medicine (INM-11), Jülich Aachen Research Alliance (JARA), Forschungszentrum Jülich GmbH, Jülich, Germany; ^7^Jülich Aachen Research Alliance (JARA)-Brain – Translational Medicine, Aachen, Germany; ^8^Department of Neurology, University Hospital RWTH Aachen, Aachen, Germany; ^9^Theoretical Systems Neurobiology, RWTH Aachen University, Aachen, Germany

**Keywords:** active vision, guided visual search, magnetoencephalography (MEG), Granger causality, generalized partial directed coherence, eye-tracking

## Abstract

In our daily lives, we use eye movements to actively sample visual information from our environment (“active vision”). However, little is known about how the underlying mechanisms are affected by goal-directed behavior. In a study of 31 participants, magnetoencephalography was combined with eye-tracking technology to investigate how interregional interactions in the brain change when engaged in two distinct forms of active vision: freely viewing natural images or performing a guided visual search. Regions of interest with significant fixation-related evoked activity (FRA) were identified with spatiotemporal cluster permutation testing. Using generalized partial directed coherence, we show that, in response to fixation onset, a bilateral cluster consisting of four regions (posterior insula, transverse temporal gyri, superior temporal gyrus, and supramarginal gyrus) formed a highly connected network during free viewing. A comparable network also emerged in the right hemisphere during the search task, with the right supramarginal gyrus acting as a central node for information exchange. The results suggest that all four regions are vital to visual processing and guiding attention. Furthermore, the right supramarginal gyrus was the only region where activity during fixations on the search target was significantly negatively correlated with search response times. Based on our findings, we hypothesize that, following a fixation, the right supramarginal gyrus supplies the right supplementary eye field (SEF) with new information to update the priority map guiding the eye movements during the search task.

## Introduction

In neurocognitive experiments, researchers generally perform multiple repetitions of the same experimental condition in a tightly controlled environment with the goal of isolating specific aspects of the cognitive process in question. An experimental setting involving simple but easily controlled sensory stimuli increases the likelihood of deducting useful knowledge from its outcome. When studying visual processing in the human brain, the high complexity of naturalistic stimuli has typically been avoided in favor of artificial and simple stimuli, such as bars, gratings, letters, or simplified scenarios like controlled saccade tasks (Yagi, 1981; Thickbroom and Mastaglia, 1985; Thickbroom et al., 1991; Kazai and Yagi, 1999; Dandekar et al., 2012; Brouwer et al., 2013), reading paradigms (Marton and Szirtes, 1988a,b; Dimigen et al., 2011) or visual search tasks with artificial stimuli (Treisman, 1982; Wolfe, 1994a).

However, while artificial stimuli make it easier to reduce the complexity of studies, there is no guarantee that responses to artificially simplified stimuli used in laboratories reflect neural responses to natural scenes (Rao et al., 2007). Indeed, there is evidence of differences between the processing of simple visual stimuli and naturalistic stimuli (Wolfe, 1994b; Vinje and Gallant, 2000; Li et al., 2003; Ossandón et al., 2010; Snow et al., 2011; Kaunitz et al., 2014; Sonkusare et al., 2019; Snow and Culham, 2021).

Compared to common experimental paradigms where participants either have to fixate on a specific point continuously or where eye movements are elicited by the appearance of a stimulus, the exploration of natural still images is driven by self-paced gaze shifts over a longer period of time (“active vision”) (Nikolaev et al., 2016). This use of unrestricted exploration of naturalistic stimuli may lead to new insights compared to experiments where eye movements are tightly controlled (Kamienkowski et al., 2012).

However, the self-paced nature of the eye movements makes comparisons across participants and/or trials difficult. A possible solution to this is to combine eye-tracking with high temporal resolution neuroimaging to obtain a comprehensive record of the visual system (Nikolaev et al., 2016). In this setup, eye-tracking provides information about where and when the participant fixates a point of interest and thus indicates which visual input is currently being processed, while neuroimaging records the response of the brain to this particular information. Eye-tracking has become an established complementary source of information for neuroimaging and has been used successfully in conjunction with electroencephalography (EEG) (Ossandón et al., 2010; Kamienkowski et al., 2012; Kaunitz et al., 2014; Seidkhani et al., 2017; Jo et al., 2019b), magnetoencephalography (MEG) (Parr et al., 2019), and functional magnetic resonance imaging (fMRI) (Jo et al., 2019a; Agtzidis et al., 2020).

The fixation-related evoked potential (FRP), i.e., the electrophysical response following a fixation, has recently been identified as a neural marker of natural visual processing (Ossandón et al., 2010; Nikolaev et al., 2016). Thus, using the FRP, it becomes possible to investigate neural activities occurring as a result of unconstrained voluntary eye movements that resemble natural visual exploration. Furthermore, cognitive processes of object identification, as well as object recognition, have been associated with the FRP (Kamienkowski et al., 2012; Kaunitz et al., 2014), suggesting that it reflects aspects of both bottom-up sensory processing and top-down cognitive processing.

To effectively interact with the environment, it is important for the brain to process information selectively in line with contextual priorities. Previous studies have reported evidence for such task-dependent selective information processing. For example, modulations of large-scale brain networks during the processing of complex visual information were highly predictive of the task (identify depicted animals vs. what the animals are doing) (Wen et al., 2019) and goal-directed behavior may result in additional top-down processes guiding visual exploration (Chen and Zelinsky, 2006). However, the way goal-directed processing of naturalistic visual stimuli affects the functional networks in the brain during active vision is still unclear.

Jo et al. (2019a,b) explored the question of how top-down and bottom-up processes during a search and a memory task affect the whole-brain connectivity network as compared to freely viewing natural images by analyzing simultaneous recordings of EEG and fMRI in two separate studies. Based on the data acquired, they found that (a) different functional structures were manifested in the visual ventral stream with visual areas V1 and V4 showing task-dependent activation; and that (b) forward connections in the ventral visual stream were enhanced during memorizing, while backward connections were enhanced during searching. However, one limitation of fMRI studies is that neural activity is reflected on slow time scales. Considering that the average duration of a fixation is as short as about 220 ms, the fast processes that occur in response to fixations would not be adequately resolved using fMRI. While the high temporal resolution of EEG alleviates this problem, it comes with a relatively coarse spatial resolution. In a dynamic causal modeling (DCM) analysis (Friston et al., 2003), Jo et al. (2019b) focused only on a small subset of regions (V1, V4, and inferior temporal gyrus), which showed significant differences in activity among the three tasks. Thus, areas with comparable activity across all tasks might have been missed. This may lead to false-positive interactions since all relevant areas need to be considered for obtaining a true representation of the network dynamics (Granger, 1980; Geweke, 1984).

In the present study, the same experimental paradigm as employed by Jo et al. (2019a,b) was used to study the change of information flow during guided visual searching as compared to the free viewing of natural scene images. The fixation-related evoked field (FRF) was recorded using MEG, enabling fast processes to be resolved with similar temporal resolution but with better spatial resolution than EEG. Following the typical MEG procedure, the FRF sensor data was transformed to the source space, yielding the fixation-related evoked activity (FRA). The analysis of FRA data was composed of three parts. First, regions of interest (ROIs) with significant FRA were identified using spatiotemporal cluster permutation testing (SCPT) (Maris and Oostenveld, 2007). Second, the activity in these ROIs was correlated with the response times during the search task. Finally, the whole-brain cortical directed connectivity was estimated using generalized partial directed coherence (GPDC) on single-epoch FRA data from these ROIs (Baccalá et al., 2007).

We hypothesize that, during a guided visual search, top-down processes occurring as a result of the specification of a search target would affect the topography of whole-brain connectivity networks. In particular, the main difference between searching and freely viewing naturalistic images would be that the visual input has to be compared to a mental representation of the search target to determine whether the input matches the target (Wolfe, 2021). Furthermore, if the search target has not been identified yet, attention has to be shifted to the next location most likely to contain the target according to a priority map (Theeuwes, 2014; Wolfe, 2021). In contrast, free viewing most likely involves attention shifts driven by bottom-up salience (Berger et al., 2012; Wolfe, 2014) as well as internal biases that reflect information currently relevant or interesting to the observer. The (right) temporoparietal junction, i.e., the border region between the superior temporal gyrus and the supramarginal gyrus, has been associated both with shifting attention to new, behaviorally relevant stimuli as part of the ventral attention network (Shulman et al., 2007; Corbetta et al., 2008) and with target detection (Linden et al., 1999; Shulman et al., 2010). Since, in response to fixations during the search task, the visual input has to be scanned for the target object and, in case the visual input does not match the target specifications, subsequent saccades to new potential target locations have to be prepared, we expect the temporoparietal junction to be a central node in the respective FRA connectivity network.

Our analysis identified two clusters of ROIs per hemisphere: one cluster in the dorsal cortex and the other in the temporoparietal cortex. These clusters exhibited significant activity during both free viewing and guided visual searching. The temporoparietal cluster consisted of parts of the posterior insula, the transverse temporal gyri, the superior temporal cortex and the supramarginal gyrus. Based on the findings from the Granger causality analysis, these four regions were highly inter-connected. In particular, one part of the temporoparietal junction, the right supramarginal gyrus, was especially well-connected during visual searching. Moreover, the correlation analysis showed that higher activity in the right supramarginal gyrus is associated with shorter response times during visual searching. Taken together, our results suggest that the right supramarginal gyrus acts as a hub for information exchange during a guided visual search.

## Materials and Methods

### Participants

Thirty-eight healthy participants between the ages of 18 and 40 took part in the study, out of which 16 were female. Seven participants were discarded due to excessive movements during the measurement, i.e., the deviation in head position at the beginning and at the end of a measurement run was greater than 2 cm. In total, 31 participants remained for analysis, out of which 14 were female.

All participants were right-handed, as assessed with a German version of the handedness questionnaire (Oldfield, 1971). Twenty-five participants had normal vision (up to ± 1 diopters), and MediGlasses for MRI made by Cambridge Research Systems (Rochester, United Kingdom) were used to correct vision to normal for the remaining six participants.

None of the participants reported a history of neurological or psychiatric diseases, as assessed by the German version of Beck’s Depression Inventory (Beck et al., 1996). After having received a full explanation of the experiment, written informed consent was obtained from the participants. The study was approved by the ethics committee of RWTH Aachen University Hospital, Germany.

### Experimental Paradigm

#### Task Protocol

**FIGURE 1:**
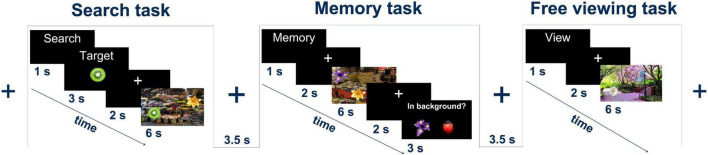
Experimental tasks: the search task, the memory task, and the free viewing task. In all tasks, a natural scene image with five foreign objects was presented for 6 s. Figure adapted from Jo et al. (2019a).

Participants performed voluntary visual exploration of still images depicting scenes of everyday life under three different task conditions: visual search (VS), memorizing (ME), and free viewing (FV) (Figure 1). Five objects were embedded in every image (cf. section “Stimulus Creation and Presentation”). At the start of each trial, a task indicator such as “Merke” (German for “memorize”), “Suche” (German for “search”), or “Schaue” (German for “look”) was presented for 1 s. A set of 30 trials per task condition was used, resulting in a total of 90 trials per participant. The trials were divided into blocks of three, with each block containing one trial for each task in a pseudo-random order to ensure sufficient variability and to ensure that the same task would not occur more than twice in a row. Between each trial, a black background with a central, white fixation cross was presented for 3–4 s. Since the focus of the present study is on the comparison of VS and FV, the data from ME was not used for any analysis.

For VS trials, following the presentation of the task indicator, a target object was displayed for 3 s, followed by a 2 s presentation of the fixation cross. A scene image was then presented for 6 s. During this time, the participants had to search the image for the previously presented target object. The participants were asked to press a button to indicate that they had found the target.

In FV trials, the task indicator was immediately followed by a 2 s display of the fixation cross. The scene image was then presented for 6 s. The participants viewed the image freely without any additional cognitive tasks.

#### Stimulus Creation and Presentation

Since the same stimuli were used as in the studies of Jo et al. (2019a,b), the stimulus creation method is described only briefly. The scene image stimuli were created by embedding object images in background images of everyday scenes. A selection of 39 object images consisting of flowers, animals, insects, fruits, furniture, tools, and vehicles was taken from the Microsoft image gallery and resized to an average of 143.56 × 141.10 pixels. The 15 background images of 1,920 × 1,200 pixels were taken from the internet, or they were taken by the co-authors of Jo et al. (2019a,b). The images were photos of plants, flowers, fruits, gardens, boats, rooms, streets, and churches. For each combination of a background and an object set, three sets of object positions were generated with the goal of letting the objects blend naturally into the background. This resulted in a total of 90 stimulus images, composed of 15 background images × 2 sets of object images × 3 sets of object positions. The three position variants of an identical object-background pair were used for the three different task types (FV, ME, and VS).

Stimulus presentation was performed using the Python package PsychoPy 2.0 (Peirce et al., 2019). A Barco FL35 WUXGA projector was used with a resolution of 1,920 × 1,200 pixels and a refresh rate of 60 Hz. All images were centered at eye level. Since the maximum visual angle of the EyeLink 1,000 Long Range eye tracker was 32 × 24°, the size of the stimulus images was adjusted to fit into that area.

### Data Acquisition

Brain activity was recorded with 248 magnetometers in blocks of 30 trials at a sampling rate of 1017.25 Hz using the Magnes 3600 WH MEG system from 4D-Neuroimaging (San Diego, United States of America). This resulted in a total of three ∼13-min MEG recording runs per participant. At the beginning and at the end of each recording run, the participant’s head position was determined. Cardiac and ocular activity were recorded at a sampling rate of 5,000 Hz using electrocardiography (ECG) and electrooculography (EOG) with the BrainAmp ExG MR system from Brain Products (Gilching, Germany). Eye movements were recorded at a sampling rate of 1,000 Hz using the EyeLink 1000 Long Range eye tracker from SR Research (Ottawa, Ontario, Canada).

At the beginning of each recording run and after every six trials, the eye tracker was calibrated using EyeLink’s 13-point calibration method (SR Research Ltd., 2009). If the average deviation was above 0.5° or the maximum deviation at one of the calibration points was higher than 2°, the calibration was repeated.

A structural MR scan was performed with a MAGNETOM Trio 3T MRI scanner from Siemens (Munich, Germany) using MPRAGE (Mugler and Brookeman, 1990). The MR scan was used for the localization of the sources in the brain that gave rise to the signal recorded at the MEG sensors.

### Data Analysis

Data analysis was performed using Python 3.6, with MNE python v0.19 being the main package for the MEG data analysis (Gramfort et al., 2013).

#### Pre-processing

After each recording session and for each of the three recording runs, the four data sets (MEG, ECG, EOG, and eye-tracking) were time-aligned and combined into single MNE python raw objects with sampling frequencies of 1017.25 Hz following resampling of the ECG, EOG, and the eye-tracking data. It is important to note that, due to potentially different head positions between recordings of the same participant, the three recording runs had to be processed separately until after the transformation to the source space for the extraction of ROI time courses (cf. section “Extraction of Regions of Interest Time Courses”).

MEG channels with strong artifacts were identified in the time and frequency domain using an in-house machine learning algorithm based on density-based spatial clustering of applications with noise (DBSCAN), as implemented in scikit-learn (Ester et al., 1996; Pedregosa et al., 2011). These channels were then replaced by a virtual channel using interpolated data from neighboring channels (Perrin et al., 1989; Gramfort et al., 2013, 2014).

Environmental noise was removed from the MEG signals by subtracting the reference signals that were recorded in parallel with the MEG signals (Robinson, 1989). Power line noise at 50 and 60 Hz noise from the projector, including their harmonics, were removed using a notch filter.

As it has been reported that filtering has a negative effect on Granger causality (Florin et al., 2010; Barnett and Seth, 2011), the analysis pipeline was split into two separate branches. The identification of ROIs with significant activity and analysis of averaged data, in general, was performed on bandpass-filtered data between 1 and 45 Hz, while the analysis of single-epoch data like the calculation of Granger causality (cf. section “Granger Causality Analysis”) was performed on unfiltered data (apart from the notch filters, which do not tend to introduce artifacts in the Granger causality analysis; Florin et al., 2010).

Independent component analysis (ICA) was used to remove components containing significant contributions of ocular or cardiac activity (Hyvärinen and Oja, 2000; Dammers et al., 2008). For this, the ∼13-min recording runs were cut into segments of approximately 100 s in length (∼100,000 samples). To avoid possible signal discontinuities, the time of the data splitting was set to be in the middle of the eye tracker calibration. To increase the reliability of the signal separation with respect to the ocular and cardiac artifacts, training of the ICA demixing matrix was performed on bandpass-filtered (1–45 Hz) data, which was then applied to both filtered and unfiltered data (Winkler et al., 2015). Following artifact removal, the cleaned data segments were concatenated to the original full-length recording run.

#### Creating Epochs

The EyeLink 1000 system detects saccades and fixations automatically in the recorded eye-tracking data. These fixation timings were used to create FRF epochs for FV and VS, with time 0 corresponding to fixation onset. For VS, fixation events after task completion, i.e., after the response was given, were ignored. The time interval of −0.2 s (for the bandpass-filtered data) or −0.4 s (for the unfiltered data) to 0 s from fixation onset was used as a baseline, i.e., the mean of the sensor time courses was calculated for that interval and subtracted from the entire epoch on a per sensor basis. The standard deviation across all channels was then computed for the baseline interval, and the sensor time courses from the entire epoch were divided by this standard deviation. Epochs for fixations during the presentation of the fixation cross were extracted in a similar fashion.

#### Identification of Regions of Interest

Based on the filtered FRF data averaged across the epochs, ROIs were identified using SCPT. The averaged FRF data from each recording run was projected from the sensor space onto the source space using dynamic statistical parametric mapping (dSPM) (Dale et al., 2000). The resulting activity at the vertices is the FRA. Afterward, the norm of the source orientations was taken (Gramfort et al., 2013).

Source space construction was performed using FreeSurfer (Dale et al., 1999; Fischl et al., 1999). For group analysis, the individual source data were morphed to FreeSurfer’s “fsaverage” common template source space with 5,124 vertices, providing an average vertex-to-vertex distance of about 6.2 mm.

To account for possible brain activity evoked by physical eye movements but not related to processing visual information during active vision, FRA during the presentation of the fixation cross was subtracted from the FRA during FV or VS. These contrasts were then used as the input for SCPT.

Two separate sets of ROIs were constructed using SCPT, one for fixation onset during FV and one for fixation onset during VS. The cluster tests were performed with 30,000 permutations based on the first 200 ms after fixation onset of the respective FRA time courses. With 31 participants and three runs, this resulted in input arrays of shape: (93 runs, 5,124 vertices, 203 time points).

To ensure that strong outliers that were very limited in either spatial or temporal extent were excluded for a given task condition, only significant clusters (*p* < 0.05) with a minimum size of five vertices (about 1.95 cm^2^) and a temporal extent of greater than 20 ms were accepted for further analysis. Since the resulting clusters were generally large and covered several anatomical areas, all remaining vertices that were part of at least one significant cluster were grouped together and then partitioned into ROIs based on the anatomical labels as defined by the Desikan-Killiany atlas (Desikan et al., 2006). If fewer than 15% of vertices in a given ROI (i.e., in an anatomical label after parcellation) were significant, the ROI was discarded from analysis.

#### Extraction of Regions of Interest Time Courses

Single-epoch source activity time courses were computed for each vertex using dSPM, with the source activity projected onto the vector normal to the cortical surface (Gramfort et al., 2013). The ROI time courses, i.e., the representative single-epoch time courses for a given ROI, were computed as the average time course of all vertices within a certain radius of the vertex with the maximum activity in the ROI.

First, the source time courses were averaged across epochs for each participant, and the vertex with the largest absolute amplitude during the main activity (i.e., between 50 and 180 ms after fixation onset) was determined for each ROI. Subsequently, the representative single-epoch time courses for a given ROI were computed by averaging the time courses of all vertices of that ROI within a radius of 12.4 mm from the vertex with maximum activity, where 12.4 mm corresponded to twice the average vertex-to-vertex distance (see section “Identification of Regions of Interest”).

However, it is important to note that, since the source activity was projected onto the vector normal to the cortical surface, the time courses of vertices at opposing sides of a sulcus are likely to have opposite signs if the direction of the underlying current is the same in both vertices. As a result, activity in these vertices would be canceled out when using a simple average. To avoid this issue, the sign of some vertices were adjusted prior to averaging. These vertices were determined using the Pearson correlation coefficient. First, the vertex time courses were averaged across epochs, and then the correlation coefficient was computed between the average time course of the vertex with maximum activity and the average time courses of the other surrounding vertices. If the correlation coefficient was negative for a given vertex, the sign of the respective single-epoch time courses was flipped.

#### Correlation Analysis Between Activity and Search Response Time

For the VS task, the FRA amplitude of each ROI was correlated trial by trial with the participant’s response time. Specifically, a Pearson’s correlation coefficient (*r*) was calculated between the bandpass filtered (1–45 Hz) single-trial FRA time series during the first fixation on the search target and the respective single-trial response times. Multiple comparisons were accounted for by controlling the false discovery rate (FDR) (Benjamini and Hochberg, 1995).

For each ROI, the full width at half maximum (FWHM) time interval was determined based on the grand average of the single-epoch FRA time courses across participants and trials, and the FRA amplitude of a given ROI was then computed as the average, absolute activity during the respective FWHM time interval. The FRA amplitudes obtained across trials were correlated with the respective response times, which were defined as the latencies from the onset of the first fixation on the search target to the participant’s response via button press.

Only trials with correct responses were included in the analysis. A response was defined as correct if, within ±500 ms of giving the response, the participant fixated within 1.7° of visual angle from the center of the target object. Furthermore, trials with response times longer than 1 s were discarded from the analysis.

#### Autoregressive Models

Autoregressive models provide the mathematical framework for the calculation of Granger causality. A set of *N* simultaneously observed time series


$$\text{X}\left(t\right)={\left[X_{1}\left(t\right),X_{2}\left(t\right),\ldots ,X_{N}\left(t\right)\right]}^{T}$$

can be represented by a multivariate autoregressive model of order *p* MVAR(*p*):


$$\text{X}\left(t\right)=\sum_{k=1}^{p}\text{A}\left(k\right)\text{X}\left(t-k\right)+\text{E}\left(t\right),$$

where the matrix of autoregressive coefficients for the *k*th time lag is given by **A**(*k*), and **E**(*t*) is a vector containing white noise error terms (Geweke, 1982, 1984). The Augmented Dickey-Fuller (ADF) and the Kwiatkowski-Phillips-Schmidt-Shin (KPSS) tests from the statsmodels Python package were used to test the recorded time series for stationarity (Kwiatkowski et al., 1992; Greene, 2003; Seabold and Perktold, 2010). Since the data was generally not stationary, first-differencing was used, which then resulted in stationary time series (Geweke, 1982; Lütkepohl, 2005; Seth, 2010; Barnett and Seth, 2011).

The MVAR analysis and the subsequent Granger causality analysis were performed using the Source Connectivity Toolbox (SCoT) (Billinger et al., 2014). The whiteness of the residuals was tested using the Li-McLeod Portmanteau (LMLP) test as implemented in SCoT (Li and McLeod, 1981; Lütkepohl, 2005). The model order of the MVAR models was determined as the minimum of the Hannan-Quinn information criterion (HQIC) (Hannan and Quinn, 1979; Lütkepohl, 2005). The consistency test enables the determination of the degree to which the correlation structure in the data was captured by the fitted MVAR models (Ding et al., 2000).

#### Granger Causality Analysis

GPDC, as implemented in SCoT, was used for the Granger causality analysis. The goal of GPDC is to identify directed interactions between pairs of time series after removing the influence of other simultaneously observed time series (Baccalá et al., 2007).

To compute GPDC, the MVAR model first has to be written in the frequency representation where the coefficient matrix is given by $\text{A}\left(f\right)=\sum_{k=1}^{p}\text{A}\left(k\right)e^{-2ifk}$ (Geweke, 1982, 1984; Ding et al., 2006; Baccalá et al., 2007). Then, GPDC from the *n*th to the *m*th signal can be calculated as:


$${\text{GPDC}}_{mn}\left(f\right)=\frac{\frac{1}{\sigma_{m}}{\bar{A}}_{mn}\left(f\right)}{\sqrt{\sum_{k=1}^{N}\frac{1}{\sigma_{k}^{2}}{\bar{A}}_{kn}\left(f\right){\bar{A}}_{kn}^{*}\left(f\right)}},$$

where σ_*m*_ is the variance of the white noise error process *E*_*m*_(*t*) for the *m*th signal and $\bar{\text{A}}\left(f\right)=\text{I}-\text{A}\left(f\right)$. Different frequency bands were defined as follows: delta (1–4 Hz), theta (4–8 Hz), alpha (8–13 Hz), beta1 (13–20 Hz), beta2 (20–30 Hz), and gamma (30–40 Hz). To obtain the GPDC values for a specific frequency band, we averaged the GPDC results across the frequencies in the band, e.g., ${\text{GPDC}}_{\alpha ,mn}=\frac{1}{6}\sum_{f=8}^{13}{\text{GPDC}}_{\text{mn}}\left(f\right)$ for the alpha band using frequency bins of 1 Hz. The GPDC values obtained constitute a causality matrix for the corresponding frequency band, with the element at the *m*th row and the *n*th column representing the strength of causal interaction from the *n*th to the *m*th signal.

#### Statistical Analysis

For each participant, GPDC was computed on the unfiltered (except for the notch filter) ROI FRA time courses in the interval of 0–300 ms from fixation onset. Only fixations that occurred prior to the participant’s response were included in the analysis of the search task. Therefore, the number of events during VS is lower than the number of events during FV. To ensure comparable SNRs for both FV and VS, the maximum number of epochs to be used for a participant was set to the minimum number of epochs across participants and conditions plus 10%. For example, if the number of epochs for one participant exceeded the threshold by 50%, every third epoch was excluded from the analysis.

To identify significant causal interactions between ROIs, surrogate data were computed for each participant. This was achieved by shuffling the data along the time axis to destroy the phase information of the ROI time courses. For each surrogate, the maximum GPDC value was identified for each frequency bin. This procedure was repeated 1,000 times per participant. The significance threshold for causal interactions in a specific frequency band was determined by averaging the 99.99th percentile of the corresponding frequency bins. Connections with a strength below the threshold were set to zero.

To determine which causal interactions were significant (*p* < 0.001) at the group level, it was assumed that number of participants for which a connection appeared with significant strength was distributed according to a binomial distribution with the number of independent trials *N* = 31 and probability *p* = 0.5. For each connection, the *p*-value was determined based on the number of participants featuring the connection.

Since the two tasks, VS and FV, have different sets of ROIs, the entries in the causality matrices are not directly comparable. To bypass this problem, the size of the causality matrices was expanded to include all ROIs that were significant during either FV or VS. GPDC values were set to zero for ROIs that were not part of the respective FV or VS network. Results were compared by subtracting the expanded VS causality matrix from the expanded FV causality matrix.

The node degree for the directed causality matrices was defined as the number of incoming and outgoing connections per ROI. To identify which ROIs had a particularly high node degree, the group level causality matrices were randomized for each condition and frequency band, and the node degree was then calculated. The procedure was repeated 1,000,000 times, from which the node degree percentiles were computed for each frequency band. All ROIs with a node degree equal to or above the 95th percentile in at least one of the frequency bands were considered for subsequent analysis.

## Results

### Behavioral and Task Performance

On average, the participants performed 21.58 ± 2.38 fixations to explore the image during the FV trials. During the VS trials, 11.80 ± 1.67 fixations were performed on average before the response, and once the search target was fixated on, it took on average 489 ± 197 ms for the participants to respond via button press.

The lowest total number of fixations during FV was 499, while the highest total number of fixations was 803. For VS, the lowest and highest total numbers of fixations were 243 and 477, respectively. Therefore, a maximum of 267 epochs (243 × 1.1 ≈ 267) was used per participant in the GPDC analysis for both tasks. The average fixation durations during FV and VS were 229.49 ± 116.64 and 201.14 ± 101.09 ms, respectively, while the saccade durations were 38.50 ± 26.52 and 41.20 ± 22.66 ms.

In VS, 75.91 ± 9.03% of search targets were identified correctly, i.e., a response was given within ± 500 ms of fixating within a 1.7° visual angle from the center of the target object. The lowest rate of successful trials was 56.67%, while the highest rate of success was 93.33%.

### Activity During Free Viewing and Guided Visual Searching

SCPT was performed on FRA time course contrasts (image presentation vs. fixation cross presentation) to identify ROIs with significant activity related to visual processing during FV and VS (see section “Identification of Regions of Interest” for details). The ROIs formed the basis for the subsequent correlation and Granger causality analyses.

The spatiotemporal clusters were generally very similar across cognitive tasks, and the activity was strongly symmetric across hemispheres. Table 1 lists the regions with significant FRA for FV and for VS, together with the Montreal Neurological Institute (MNI) coordinates of the vertices with the largest activity in the respective regions. These coordinates were used as the basis for the construction of the ROI time courses (see section “Extraction of Regions of Interest Time Courses”).

**TABLE 1:** Regions of interest (ROIs) in the left and right hemispheres showing significant differences in activity between events during the image presentation and events during the presentation of the fixation cross.

ROI	#	Anatomical label	Cluster location			
			Free viewing (FV)		Visual search (VS)	
			Left hemi.	Right hemi.	Left hemi.	Right hemi.
**Frontal lobe**						
ParaCeL	1	Paracentral lobule	DC −17, −31, 43	DC 18, −42, 43	–	DC 18, −42, 43
**Parietal lobe**						
SPC	2	Superior parietal cortex	DC −8, −90, 25	DC 14, −81, 32	DC and IPSC −8, –90, 25	DC and IPSC 14, −81, 32
IPC	3	Inferior parietal cortex (angular gyrus)	DC −29, −81, 13	DC 33, −75, 18	DC and IPSC −29, −81, 13	DC and IPSC 40, −67, 13
**SMG**	**4**	**Supramarginal gyrus**	**TPC** −**36,** −**36, 18**	**TPC 40,** −**36, 18**	–	**TPC 40,** -**36, 18**
PreC	5	Precuneus	DC −21, −61, 9	DC 20, −56, 9	DC −21, −61, 9	DC 17, −55, 14
**Temporal lobe**						
**STG**	**6**	**Superior temporal gyrus**	**TPC** −**38,** −**37, 11**	**TPC 40,** −**35, 13**	–	**TPC 1,** −**16, 23**
MTG	7	Middle temporal gyrus	–	DC 49, −59, 6	–	DC 49, −59, 6
ITG	8	Inferior temporal gyrus	DC −43, −53, −10	DC 45, −63, −9	DC −43, −53, −10	DC 45, −63, −9
BSTS	9	Banks superior temporal sulcus	DC −44, −54, 9	DC 46, −46, 10	DC −48, −53, 6	DC 46, −46, 10
FG	10	Fusiform gyrus	DC −29, −74, −7	DC 27, −74, −7	DC −29, −74, −13	DC 27, −74, 7
**TTG**	**11**	**Transverse temporal gyri**	**TPC** −**38, −27, 5**	**TPC 44,** −**21, 5**	**TPC** −**41, −26, 2**	**TPC 44,** −**21, 5**
EC	12	Entorhinal cortex	DC −20, −10, −30	DC 21, −11, −29	EPC −22, −22, −23	EPC 21, −11, −29
PHG	13	Parahippocampal gyrus	DC −20, −18, −26	DC 23, −17, −28	EPC −20, −18, −26	EPC 23, –17, −28
**Occipital lobe**						
LOC	14	Lateral occipital cortex	DC −16, −97, −2	DC 30, −86, 1	DC −16, −97, –2	DC 30, −86, 1
LG	15	Lingual gyrus	DC −11, −85, −13	DC 30, −86, 1	DC −11, −85, −13	DC 17, −62, 1
C	16	Cuneus	DC −10, −73, 19	DC 10, −79, 28	DC −10, −73, 19	DC 10, −79, 28
PeriCC	17	Pericalcarine cortex	DC −15, −94, 1	DC 18, −94, 0	DC −15, −94, 1	DC 18, −94, 0
**Cingulate cortex**						
PCC	18	Posterior cingulate cortex	DC −16, −37, 39	DC 11, −38, 41	DC −2, −23, 27	DC 5, −28, 27
ICC	19	Isthmus cingulate cortex	DC −14, −51, 4	DC 17, −50, 4	DC −14, −51, 4	DC 17, −50, 4
**Insula**						
**PI**	**20**	**Posterior insula**	**TPC** −**35,** −**20, 1**	**TPC 35,** −**19, 11**	**TPC** −**35,** −**20, 1**	**TPC 34,** −**19, 7**

*The clusters (DC, dorsal cluster; TPC, temporoparietal cluster; IPSC, intraparietal sulcus cluster; EPC, entorhinal-parahippocampal cluster) and the MNI coordinates from the vertices with maximum activity are given for each ROI. “-” indicates the absence of significant activity. The four regions belonging to the temporoparietal cluster are marked in bold.*

**FIGURE 2:**
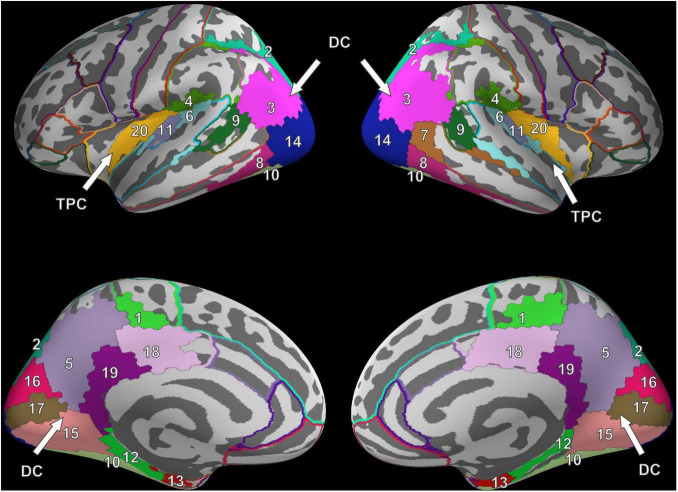
Cluster results for the fixation onset contrast of image presentation during FV vs. fixation cross presentation. Separate ROIs according to the Desikan-Killiany atlas are highlighted in different colors (Desikan et al., 2006). The identified clusters (DC, dorsal cluster; TPC, temporoparietal cluster) are mostly symmetric across hemispheres. Corresponding ROI names are listed in Table 1.

For FV, two clusters were found in each hemisphere and were noted to be at similar locations when compared across hemispheres. The first cluster was located in the posterior part of the brain, covering the occipital lobe, parts of the parietal lobe, the temporal lobe, and large parts of the cingulate cortex (Figure 2). In the following, this cluster is referred to as the dorsal cluster. The second cluster covered areas of the supramarginal gyrus (#4), the superior temporal gyrus (#6), the transverse temporal gyri (#11), and the posterior insula (#20) and is referred to as the temporoparietal cluster. The middle temporal gyrus (#7) was the only region that was present in the right hemisphere only.

**FIGURE 3:**
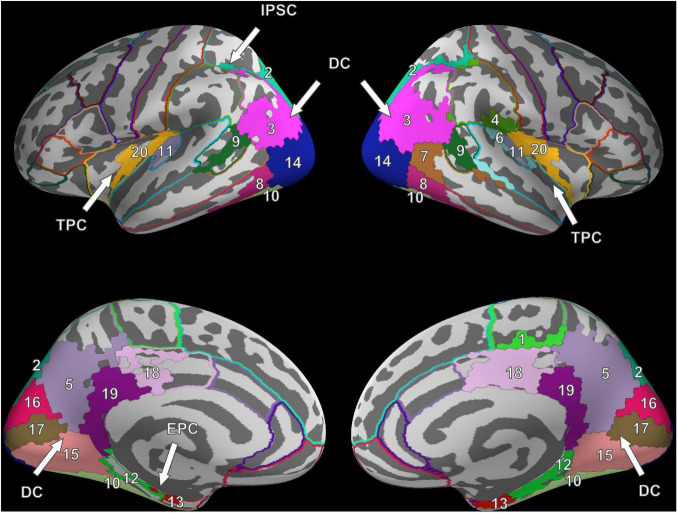
Cluster results for the fixation onset contrast of image presentation during VS vs. fixation cross presentation. Separate ROIs according to the Desikan-Killiany atlas are highlighted in different colors (Desikan et al., 2006). The identified clusters (DC, dorsal cluster; TPC, temporoparietal cluster; IPSC, intraparietal sulcus cluster; EPC, entorhinal-parahippocampal cluster) are mostly symmetric across hemispheres. Corresponding ROI names are listed in Table 1.

Six clusters were found for VS and were at roughly the same locations as compared to the clusters found during FV. However, while the dorsal cluster covered one contiguous area in the left hemisphere during FV, the cluster was split into three separate clusters during VS, one large and two considerably smaller ones. One of the smaller clusters covered parts of the entorhinal cortex (#12) and the parahippocampal cortex (#13) in the left hemisphere (cf. bottom left of Figure 3). Therefore, it is referred to as the entorhinal-parahippocampal cluster. The other small cluster covered small parts of the superior parietal cortex (#2) and the inferior parietal cortex (#3) at the intraparietal sulcus (cf. top left of Figure 3) and is therefore referred to as the intraparietal sulcus cluster. Compared to FV, several regions, namely the paracentral lobule (#1), supramarginal gyrus (#4), and superior temporal gyrus (#6), exhibited significant FRA in the right hemisphere during VS but not in the left hemisphere. Notwithstanding the paracentral lobule, no significant activity was found in other frontal areas.

**FIGURE 4:**
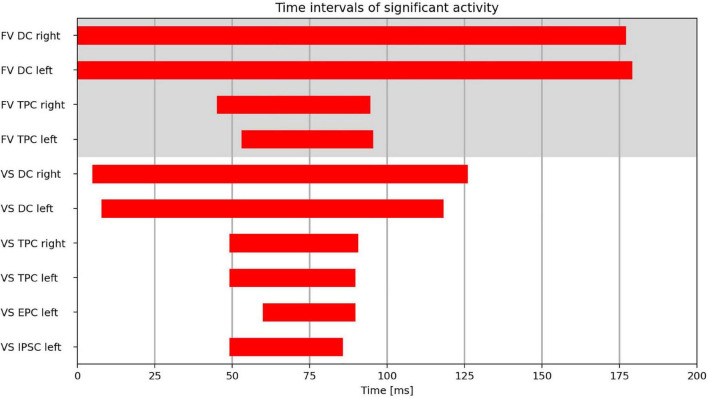
Time intervals with significant activity in the cluster regions (DC, dorsal cluster; TPC, temporoparietal cluster; IPSC, intraparietal sulcus cluster; EPC, entorhinal-parahippocampal cluster) during FV and VS with fixation onset at *t* = 0 ms.

The time intervals during which the clusters exhibited significant activity can be found in Figure 4.

### Search Response Time and the Right Supramarginal Gyrus

In order to identify the ROIs involved in processing visual information related to the search target, the relationship between the activity recorded during the first fixation in a trial on the search target and the associated response in that trial was analyzed. For this, the Pearson correlation was calculated between the single-trial FRA amplitudes and the single-trial response times (see section “Correlation Analysis Between Regions of Interest Fixation-Related Evoked Activity Amplitude and Response Time**”** for details).

**FIGURE 5:**
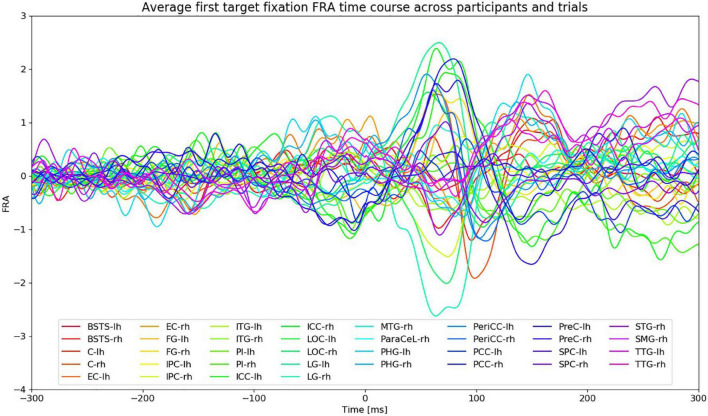
FRA time courses of the first fixation on the search target averaged across all VS trials and participants. Fixation onset is at 0 ms. “lh” and “rh” stand for left and right hemisphere, respectively. Time courses from different ROIs are color-coded.

Figure 5 shows the FRA time series of the first fixation on the search target averaged across participants and VS trials. The amplitudes of the ROI time courses start to rise at around 30 ms after fixation onset. Given the average fixation duration during VS trials (section “Behavioral and Task Performance”), the peaks after 200 ms from fixation onset are most likely the result of subsequent saccades and fixations. The FWHM time intervals were therefore restricted to the time between 30 and 200 ms from fixation onset.

**FIGURE 6:**
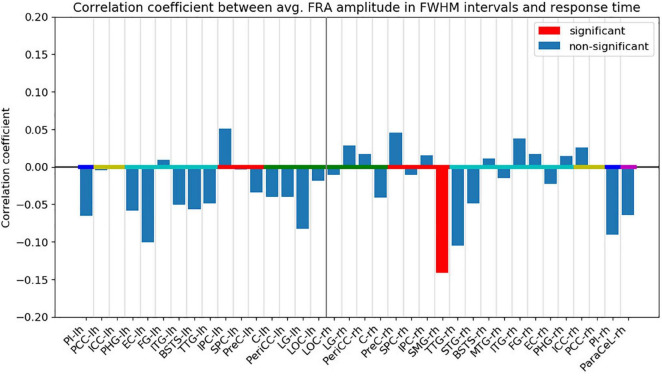
Pearson’s correlation coefficient between the absolute ROI time course FRA amplitude averaged across the FWHM time intervals and the response time. Red bars indicate that the correlation coefficient was significant (*p* < 0.05) after FDR-correction. The ROIs are ordered by hemisphere and anatomical higher-level regions (frontal lobe: magenta, insula: blue, cingulate cortex: yellow, temporal lobe: turquoise, parietal lobe: red, occipital lobe: green). “lh” and “rh” stand for left and right hemisphere, respectively.

Following FDR-correction, a significant, negative correlation (*r* = −0.141, *p* < 0.05) was found between the response time and the FRA amplitude in the right SMG (Figure 6). The FWHM time interval for the right SMG ranged from 113 to 181 ms from fixation onset (see Supplementary Material). The negative correlation indicates that the response times tended to be shorter for trials with higher activity in the right SMG. Other ROIs did not exhibit significant correlations.

### Granger-Based Causal Interactions

In order to analyze how top-down guidance during VS influences the network topology, GPDC was used to compute the whole-brain effective connectivity networks for FV and VS based on the respective ROI time courses (see section “Granger Causality Analysis” for details).

After first-differencing, the ROI time courses were weakly stationary according to the ADF and KPSS tests (*p* < 0.05). Using the HQIC, the optimal MVAR model order was estimated to be 42. Furthermore, using SCoT, it was confirmed that the resulting MVAR models represented stable (i.e., stationary) processes and that the residuals could be considered white according to the LMLP test (Billinger et al., 2014). According to the consistency test, approximately 76% of the correlation structure in the data was captured by the fitted MVAR models.

**FIGURE 7:**
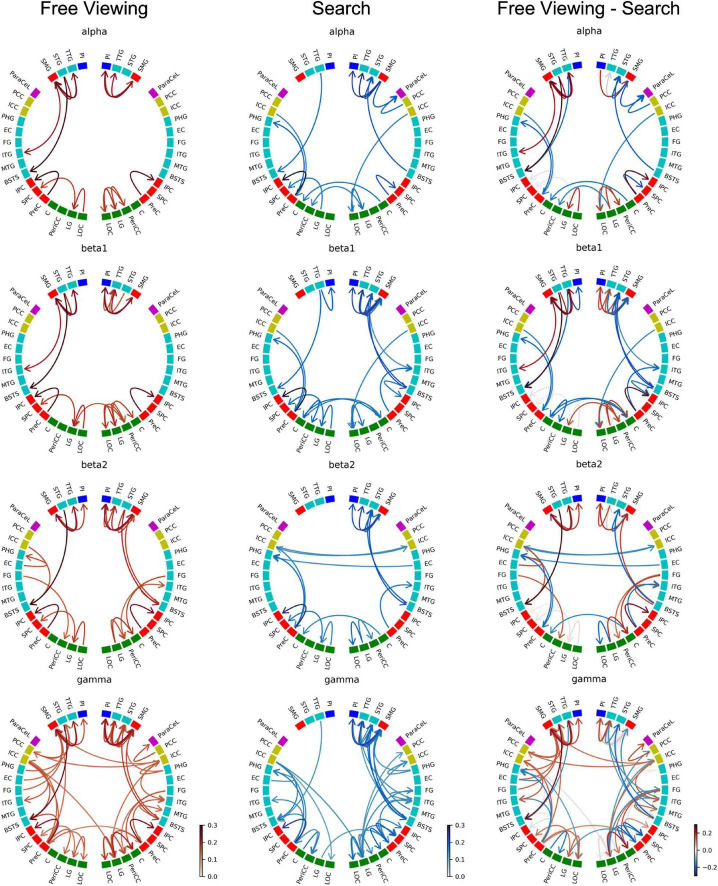
Causality group results (GPDC) for fixation onset during FV, VS, and the direct comparison between FV and VS where the causality matrix containing the group results for VS was subtracted from the causality matrix containing the group results for FV. Red arrows indicate that the connection was stronger during FV, while blue arrows indicate that VS dominated. Gray arrows indicate that the connections were of comparable strength during FV and VS. The ROIs are ordered by hemisphere and cluster (TPC and DC). The color of the nodes indicates the anatomical higher-level region (frontal lobe: magenta, insula: blue, cingulate cortex: yellow, temporal lobe: turquoise, parietal lobe: red, occipital lobe: green). Results from the delta and theta band can be found in Supplementary Material.

Figure 7 depicts the GPDC group results for FV, VS, and the contrast between FV and VS. The ROIs were grouped by brain section: frontal lobe (magenta), insula (blue), cingulate cortex (yellow), temporal lobe (turquoise), parietal lobe (red), and occipital lobe (green). Since the connections in the alpha band contain all the connections found in the delta and theta band, here we present the results for the alpha and higher frequency bands only. Results for the delta and theta band can be found in Supplementary Material.

As shown in Figure 7, fixations during FV mainly led to interactions within the parietal, temporal, and occipital regions. In particular, these interactions included visual areas in the occipital regions and in the (inferior) temporal cortex. This pattern was observed in all frequency bands.

**FIGURE 8:**
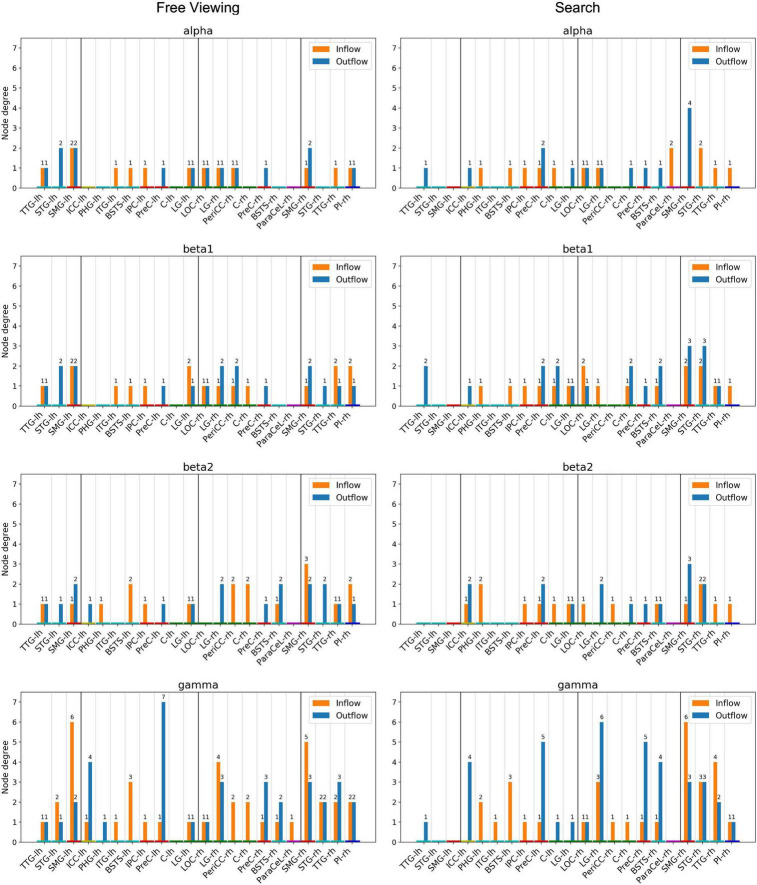
Node degree, i.e., the number of incoming and outgoing connections per ROI. Only ROIs with a node degree equal to or above the 95th percentile in at least one frequency band for either task were included. The ROIs are ordered by hemisphere and cluster (TDC and DC). The color of the nodes indicates the anatomical higher-level regions (frontal lobe: magenta, insula: blue, cingulate cortex: yellow, temporal lobe: turquoise, parietal lobe: red, occipital lobe: green). Results from the delta and theta band can be found in Supplementary Material.

There was high local connectivity between the neighboring regions in the temporoparietal cluster in both hemispheres (cf. TPC in Figure 2 consisting of the PI, SMG, STG, and TTG). Moreover, these temporoparietal cluster regions were also well-connected with other areas in the temporal lobe (BSTS, EC, FG, ITG, and PHG), the parietal lobe (PCC and PreC), and the ICC in the cingulate cortex. This was reflected by the high node degree of these four regions (cf. Figure 8). Generally, the number of connections involving these regions increased concomitantly with the increase in frequency. Similarly, there were many connections involving regions of the occipital lobe. However, there were only five inter-hemispheric causal interactions, specifically, from the left ICC to the right LG, from the right ICC to the left PCC and to the left SMG, from the right PeriCC to the left LG, and from the left PreC to the right PreC.

Figure 7 depicts the GPDC group results for fixation onset during VS where similar to FV the number of connections generally increased along with the increase in frequency. Also similar to FV, interactions were mainly observed within the parietal, temporal, and occipital regions.

In the right hemisphere, regions belonging to the temporoparietal cluster were again seen to be well-connected. Connections with the temporoparietal cluster involved regions in the temporal lobe (BSTS, ITG, MTG, PHG) and the ParaCeL in the frontal lobe. Out of the four ROIs in the temporoparietal cluster, the right SMG particularly stood out as having the highest number of connections with other areas and was connected with all of the regions mentioned above, in addition to the other temporoparietal cluster regions. The high local degree of connectedness of the temporoparietal cluster was absent in the left hemisphere. The only inter-hemispheric causal interactions were from the right C to the left C and vice versa, the right EC to the left PHG, the left ICC to the right ICC and vice versa, from the left ICC to the right LG, from the left LG to the right PreC, and from the right LG to the left PeriCC.

For both tasks, the majority of the intra-sectional connections and even many of the inter-sectional connections were short-range connections, i.e., the majority of interactions took place between neighboring ROIs during both tasks. Few regions featured directed interactions across hemispheres.

For a direct comparison of GPDC results for FV and VS, the causality matrix for VS was subtracted from the causality matrix for FV (cf. Figure 7). From the circular plots, it becomes evident that most connections were not shared between the two conditions. Only a few connections, e.g., the right SMG to the right PI, the right SMG to the right TTG, or the right PI to the right STG, showed consistent strengths during both tasks (gray arrows). Compared to FV, the local interactions between the temporoparietal cluster regions in the right hemisphere were strongly enhanced in VS.

For each task, random permutation analysis revealed several ROIs with a high node degree in at least one of the frequency bands (Figure 8). Twelve regions showed a high node degree during FV. The node degree was significantly high for the SMG, TTG, and LG in both hemispheres while the ITG, PreC, and STG were only significant in the left hemisphere and the BSTS, the PI, and the PeriCC were highly connected in the right hemisphere. For VS, there were 16 regions with a large number of connections. The BSTS, C, and PreC exhibited a high node degree in both hemispheres, while the four temporoparietal cluster regions only showed a large number of connections in the right hemisphere. Moreover, the LOC, LG, and ParaCeL were identified to be well-connected within the right hemisphere, while the ICC, IPC, and PHG were the only ROIs with a high node degree solely within the left hemisphere.

The high local degree of connectedness observed between temporoparietal cluster regions during FV was absent in the left hemisphere during VS. At the same time, the right SMG had a higher node degree during VS as compared to FV across the majority of frequency bands (Figure 8, Search). Indeed, the highest number of connections for a single ROI was found in the right SMG in the gamma band during VS, with six incoming and three outgoing connections. The only other region with a total of nine connections was the right LG during VS, with three incoming and six outgoing connections in the gamma band. For VS, the next highest total number of connections was six, which was found in the right TTG, the right STG, and in the PreC in both hemispheres. There tended to be fewer connections per ROI in the left hemisphere during VS as compared to the right hemisphere. The highest number of connections in the left hemisphere was found in the PreC, with five outgoing and one incoming connection in the gamma band. In a given frequency band, the node degree of the right SMG during VS was higher or equal to the node degree of any other region during VS or FV.

For FV, the highest total number of connections was eight, which was found in the gamma band in the SMG in both hemispheres and in the left PreC (Figure 8, Free Viewing). The left SMG had six incoming and two outgoing connections, while the right SMG had five incoming and three outgoing connections. Seven outgoing connections were identified for the left PreC, while only one incoming connection was identified. The next highest total number of connections was seven, which was found for the right LG in the gamma band, with four incoming and three outgoing connections. In the majority of frequency bands, the left SMG had the largest number of connections during FV.

## Discussion

Using SCPT, ROIs with significant FRA were identified during the processing of visual information, which was gathered during naturalistic free viewing and search tasks through self-paced eye movements. For both tasks, a temporoparietal and a dorsal cluster of ROIs were identified in each hemisphere and were shown to be highly symmetric across hemispheres. The causal interaction network revealed by Granger causality analysis using GPDC showed a high degree of connectedness between the temporoparietal cluster regions, but also with other ROIs during FV. The right SMG was found to be especially well connected during VS and was also the only ROI in which activity was significantly correlated with shorter response times during VS. These results support the hypothesis that the whole-brain connectivity is affected by goal-directed behavior and accounts for the additional processes required for a guided visual search.

### Regions of Interest

Thirty-nine ROIs (19 in the left and 20 in the right hemisphere) were identified for FV and 36 (16 in the left and 20 in the right hemisphere) for VS (Table 1). The ROIs in the two hemispheres were highly symmetric, and the 20 ROIs in the right hemisphere were found to be identical in FV and VS. However, a major difference between the two tasks was that three ROIs in the left hemisphere, namely the SMG, STG, and ParaCeL, were only significantly active during FV and not during VS.

The temporoparietal junction is known to comprise parts of the SMG and STG, and several authors have reported that damage in the right temporoparietal junction causes visual processing defects, such as left spatial neglect or hemiextinction (Karnath et al., 2004; Meister et al., 2006; Corbetta et al., 2008; Ticini, 2013). Shulman et al. (2007) and Corbetta et al. (2008) proposed that the right temporoparietal junction, together with the right ventral frontal cortex, forms a ventral attention network involved in redirecting attention to new objects that are behaviorally relevant. Since the right SMG and STG were found to be active during both FV and VS in our study, this may indicate that attention shifting engages similar mechanisms when guided by bottom-up salience and top-down processes.

However, significant activity was also seen in the left temporoparietal junction during FV while being absent during VS. A potential reason for this difference would be the differential response of the left and right temporoparietal junction to target and non-target (distractor) stimuli. It has been reported that the left temporoparietal junction responds only to distractor stimuli in the right hemifield, while the right temporoparietal junction responds to distractors in both hemifields (Dragone et al., 2015; Silvetti et al., 2016). At the same time, it was found that targets engage the left and right temporoparietal junction, irrespective of the hemifield they appear in. This could explain the absence of significant activity in the left temporoparietal junction during VS in our study: since, in our experiment, non-target stimuli appeared equally in the left and right hemifields, the left temporoparietal junction might have been activated only during about half of the fixations, thus leading to the activity being much weaker in the left temporoparietal junction than in the right when averaged across all fixations. This would not apply to the activity during FV because there was no distinction between target and non-target stimuli in FV. Further analysis of the FRA considering the location of target and non-target stimuli in relation to the fixation position would elucidate the suggested influence of stimulus locations on the lateral asymmetry of the SMG and STG activation during VS.

The ParaCeL, which was also absent for VS in the left hemisphere, was shown to encompass the supplementary eye field (SEF), which is, along with the frontal eye field and the parietal eye field, one of the three main areas in primates dedicated to the execution of eye movements (Grosbras et al., 1999). Electrical stimulation of the SEF was found to elicit eye movements in macaque monkeys (Schlag and Schlag-Rey, 1987, 1992; Tanji, 1994; Tehovnik, 1995; Grosbras et al., 1999), and the SEF was seen to be active during voluntary, self-paced eye movements in humans (Petit et al., 1993). Several studies have noted a left-hemispheric dominance of the SEF, particularly when endogenous eye movements were being performed (Petit et al., 1993; Grosbras et al., 1999). At the same time, the engagement of the right SEF during visually guided saccadic eye movements has also been reported (Luna et al., 1998). Taken together, these observations could explain the activity seen in the SEF during FV and VS in our study, as endogenously generated eye movements due to bottom-up salience during FV could depend more strongly on a left-hemispheric network as compared to top-down guidance during VS.

The ROIs were found to form two major clusters in each hemisphere: the temporoparietal cluster and the dorsal cluster. The fact that the PI and the TTG formed the temporoparietal cluster together with the SMG and the STG indicates existing functional relationships between these areas. Activations in the insula have been observed in a multitude of tasks (Craig, 2009; Kurth et al., 2010), although in our study, only the posterior parts of the insula were significantly active in response to fixations during FV and VS. While the PI has often been associated with empathy, pain, and somatosensory stimuli (Kurth et al., 2010), hemispatial neglect has been reported in patients with cortical damage in the PI (Golay et al., 2008). Furthermore, connections with posterior temporal, parietal, and sensorimotor areas have been found, which is mostly in agreement with the other regions in the temporoparietal cluster, i.e., the STG, TTG, and SMG (Cloutman et al., 2012; Ghaziri et al., 2017; Uddin et al., 2017). The TTG, also known as Heschl’s gyri, are generally considered to be the location of the primary auditory cortex (Morosan et al., 2001; da Costa et al., 2011); nonetheless, there is some evidence that they might also be involved in visual processing. For example, it has been reported that intraoperative electrical stimulation (Gharabaghi et al., 2006) and transcranial magnetic stimulation (Ellison et al., 2004) of the TTG lead to a disturbance of visual search behavior. Furthermore, in patients with spatial neglect, Karnath et al. (2001) found overlap in lesions in the right temporal lobe in Brodmann areas 22 and 42, the latter of which is part of the TTG (Campain and Minckler, 1976). Based on our finding that the SMG, STG, TTG, and the PI formed a cluster, and the fact that, according to the literature, damage to any of these regions may result in spatial neglect, we argue that these ROIs form a functional network. This notion is further supported by the results of the causal interaction analysis (see section “Granger-Based Causal Interactions”), where a large number of interactions were identified between these ROIs.

In addition to the temporoparietal cluster, SCPT also revealed significant activity in the dorsal regions of the brain during both FV and VS, forming the dorsal cluster. According to the probabilistic maps of visual areas created by Wang et al. (2015), we see that the primary parts of the visual cortex are located in the C, PeriCC, and LG. The ventral stream flows from the LG to the FG, and the dorsal stream goes from C through the LOC to the IPC and from there on toward the MTG and the ITG (Goodale and Milner, 1992; Milner and Goodale, 2006; Wang et al., 2015). Thus, the dorsal cluster encompasses the primary and many of the higher visual areas. Strong FRA in the dorsal cluster ROIs in both tasks indicates that these areas are involved in visual processing mechanisms common to both FV and VS. These may include the extraction of basic features like color, contrast or orientation, and the detection of boundaries (Zeki et al., 1991; Lee et al., 1998; Tootell et al., 1998), and may also involve the calculation and integration of feature-specific salience maps into combined supra-dimensional activation maps (Found and Müller, 1996, 1997; Weidner and Müller, 2009; Liesefeld and Müller, 2021; Wolfe, 2021).

Despite clear differences in the processing requirements for FV and VS, the areas covered by the clusters were mostly identical in both tasks. Furthermore, if the ROI identification is performed using the contrast of the average FRAs between FV and VS, no significant clusters were found (results not shown). Consequently, potential differences in the averaged time courses between the two tasks were small, possibly indicating that important differences on a single-epoch level were lost in the averaging process. Indeed, the single-epoch Granger causality analysis revealed differences in the causality networks between the two tasks (see section “Granger-Based Causal Interactions”). This suggests that, although mostly the same ROIs are being engaged, the way in which these ROIs interact with one another is crucial for understanding the difference between free viewing and guided visual searching.

In summary, literature evidence indicates that all four areas of the temporoparietal cluster are involved in visual processing and/or attention shifting and not just the temporoparietal junction (SMG and STG). It has been observed that damage to these areas or inactivation, e.g., through TMS, can result in defects in visual processing. Since significant FRA was seen during both FV and VS, it is likely that the temporoparietal cluster plays an important role in visual search and visual exploration. Finally, when comparing the two tasks, the overall differences between the areas with significant FRA were small, suggesting that the same areas are required for performing search tasks and when freely viewing images. We therefore hypothesize that the important distinctions only lie in how these areas interact with one another during each task.

### Fixation-Related Evoked Activity and the Search Response Time

To relate the neuronal activity to behavioral performance during VS, we examined the correlation between the FRA amplitudes for the first fixation on the target object and the subjects’ response times on single trials. A significant, albeit rather weak, negative correlation was found between the FRA amplitude in the right SMG and the response time, indicating that stronger activity in the right SMG is associated with shorter response times. No significant correlation was found for any other ROIs.

The FRA in the right SMG exhibited the largest amplitude in the time interval between 113 and 181 ms from fixation onset, and the activity in this time period was significantly correlated with the response time. Previous studies have indicated that representations of complex visual objects would already have been established in the visual system before this time interval. For example, in an experiment on macaque monkeys performing a rapid face categorization task, discrimination between different stimuli is possible after, on average, 108 ms from stimulus onset based on single-unit activity in the anterior superior temporal sulcus (Keysers et al., 2001). Since humans and macaques demonstrated similar performances in rapid categorization (Keysers et al., 2001), the visual systems for these two species likely operate on similar timelines. These findings indicate that low-level visual features, such as color and orientation, would have already been processed before the FWHM time interval in our experiment. Therefore, we argue that higher-level processes are more likely to explain the observed correlation.

First, according to the feature integration theory proposed by Treisman and Gelade (1980), an attention-related process initially binds different visual features into representations of objects (Treisman and Gelade, 1980; Treisman, 1996; Cave and Wolfe, 1999; Roskies, 1999). As the right SMG has previously been associated with such attention-related processes (Shulman et al., 2007, 2010; Corbetta et al., 2008), this might explain the observed negative correlation; greater attention, accompanied by larger amplitudes in the right SMG could lead to faster integration of visual features and hence to faster target recognition. Second, it has been reported that the right SMG facilitates trans-saccadic integration, which requires object features and locations to be bound and updated across saccades (Dunkley et al., 2016). Similar to the feature binding mentioned above, this is also a process modulated by attention (Stewart and Schütz, 2018) and, as such, greater attention reflected by stronger activity in the right SMG could lead to faster trans-saccadic integration and faster target recognition. Finally, another possible explanation is the process of template matching. According to Wolfe’s Guided Search 6.0, visual input needs to be matched against a template of the target object in order to determine if the target has been found (Wolfe, 2021). Since this process would be directly related to the speed of target recognition and hence to the response time, the observed negative correlation may indicate the involvement of the right SMG in this process. All three proposed processes are related to object recognition and are thus in agreement with previous findings that the right SMG is involved in target detection (Linden et al., 1999; Shulman et al., 2010).

Of the three possible interpretations for the observed negative correlation between the FRA in the right SMG following the first fixation on the search target and the response time (binding, trans-saccadic integration, and template matching), both binding and trans-saccadic integration are processes modulated by attention. Since the SMG has previously been associated with attention-related processes, we believe these two processes offer a more likely explanation than template matching. The results support previous findings that the right SMG participates in target detection.

### Task-Dependent Causal Interactions Between Regions of Interest

We hypothesized that the processes required for a guided visual search would affect the topography of the whole-brain causal interaction network. To test this hypothesis, causal interactions between ROIs in the time interval between 0 and 300 ms from fixation onset were computed for FV and VS using GPDC. Based on random permutations, ROIs with significant node degrees were identified. The obtained causal interaction networks and node degrees indeed exhibited distinct properties depending on the task, as discussed in detail below.

For FV, ROIs in the temporoparietal cluster (PI, STG, TTG, and SMG) exhibited a large number of local causal interactions. The node degrees of all these ROIs, with the exception of the left PI, were significantly high in both hemispheres. This is consistent with previous findings from Dragone et al. (2015) and Silvetti et al. (2016) showing that rather than there being a right hemispheric dominance (cf. Corbetta et al., 2008; Shulman et al., 2010), temporoparietal areas in both hemispheres are involved in attention shifting. Our results further suggest that, in addition to the temporoparietal junction initially identified by Corbetta et al. (2008), the (right) PI and the TTG may also play a role in shifting attention.

For VS, the highest total node degree was always found in the right SMG throughout the frequency bands (Figure 8), and many of the contributing connections were only present during VS (Figure 7). Moreover, of the four ROIs in the temporoparietal cluster, the right SMG was the only ROI bidirectionally connected to all the other ROIs in the cluster, suggesting that the right SMG might play a central role within this cluster. The node degrees of the other ROIs were generally comparable to their respective degrees during FV. This enhanced connectivity in the right SMG during VS as compared to FV implies the active involvement of this area in the processes required specifically for VS.

According to our hypothesis, two possible mechanisms could explain the increased connectivity in the right SMG. First, if one compares the process of searching an image to that of freely viewing an image, an obvious difference is that searching requires a comparison of the visual information sampled at a fixation point with the mental image of the search target in order to recognize the search target. This is a cognitive process sometimes referred to as target template matching (Wolfe, 2021). Second, searching also requires a process of overt attention shifting, or in other words, saccade orientation such that the saccade following the current fixation is oriented to the location most likely to contain the target object. According to Guided Search 6.0, this process is driven by a priority map, which is an amalgamation of top-down (search template driven) and bottom-up (salience) guidance combined with other factors such as the history of prior attention or the structure and meaning of scenes (Wolfe, 2021). These two processes, target recognition and attention shifting, need not be mutually exclusive, and indeed the right SMG has been associated with both (Shulman et al., 2007, 2010; Corbetta et al., 2008). Please note that the history of prior attention is unlikely to induce systematic changes in our current findings. In principle, selection history can affect visual search in various ways. For example, a spatial bias toward locations that are more likely to contain the target can build up during the course of the experiment (Chun and Jiang, 1998) and similarly, participants can learn to ignore irrelevant and distracting information frequently occurring at specific locations (Sauter et al., 2018, 2021; Zhang et al., 2021). However, as the combination of image, objects, and object positions was unique in every trial in this experiment, selection history is unlikely to have affected the results. A further aspect of selection history involves the short-term effect of previously attended locations being less likely to be attended again within a short period of time as illustrated in the inhibition of return (e.g., Klein, 2000), and it has controversially been discussed whether or not inhibition of return plays a role in visual search (Horowitz and Wolfe, 1998; Peterson et al., 2001). Notwithstanding this point, potential inhibition of return effects are unlikely to play a significant role in the current experiment since a potential tendency to favor previously unattended locations should be similarly present in the FV and the VS condition.

In relation to the process of attention shifting, there was one intriguing causal interaction that was only present during VS, i.e., the connection from the right SMG to the right ParaCeL, the location of the SEF (section “Regions of Interests”). This connection was found in the delta, theta, and alpha bands. The SEF has been reported to play a critical role in controlling the saccade direction in the face of several competing options (Parton et al., 2007). It has also been suggested that in goal-directed behavior, the SEF computes and evaluates the expected outcome of existing saccade options for the selection of the optimal course of action (Stuphorn, 2015). These studies strongly indicate the involvement of the SEF in the generation and evaluation of the priority map for saccade guidance. In a functional connectivity study using the right SMG as seed, Baltaretu et al. (2020) found a connection between the right frontal eye field (FEF) and the left SEF when participants were performing saccades during a planned grasping motion. They concluded that FEF and SEF provide the right SMG with the saccade-related information necessary for trans-saccadic updating of grasp orientation (Baltaretu et al., 2020). In contrast, our results rather indicate the causal interaction in the opposite direction, i.e., from the right SMG to the right SEF. Since previous reports suggest, as mentioned above, that the SEF is involved in the generation and evaluation of the priority map and that the SMG plays a role in high-level integration of visual input, this connection could be interpreted as the right SMG providing the right SEF with the information necessary to update the search priority map based on the current fixation in order to determine the optimal location of the subsequent fixation. The fact that this connection was only present in lower frequency bands, which roughly correspond to the frequency of the saccade-fixation cycle (approx. four saccades and fixations per second) supports this interpretation.

Most of the observations discussed above do not show clear differences between the examined frequency bands. Hence, it is difficult to make statements regarding which functions are particularly associated with which frequencies. Moreover, several of the discussed connections are present in all frequency bands. This might mean that the communication involving the ROIs in the temporoparietal cluster is overall enhanced during VS, without specific enhancement of a particular function.

In summary, our results uphold the hypothesis that the temporoparietal junction, i.e., the SMG and STG, participate in recognizing the target and guiding eye movements during visual search. A novel observation in the present study is that the PI and the TTG are also potentially relevant for these processes. Based on the strong connections within the right temporoparietal cluster during VS, we further hypothesize that the ROIs in this cluster form a functional unit for these processes, with the SMG being a central node. We highlight the connection from the right SMG to the right ParaCeL to be of particular importance for guiding eye movements according to a search priority map.

## Conclusion

To the best of our knowledge, this study is the first to analyze the directed interactions of MEG signals in the context of natural vision by combing SCPT with Granger-based GPDC. We found a temporoparietal cluster with significant fixation-related evoked activity consisting of the PI, TTG, STG, and SMG, as well as a dorsal cluster composed of the occipital area (primary visual areas) and areas associated with the ventral and dorsal visual streams (higher visual areas). GPDC analysis revealed these areas in the temporoparietal cluster to be tightly connected with each other as well as with areas outside the cluster during both the FV and the VS task. Our results point toward these regions being involved in guiding eye movements during active vision. In particular, the right SMG exhibited the highest degree of causal interactions during the search task, indicating its central role in guided visual searching. We specifically note that the interaction from the right SMG to the right SEF would suggest that the former provides the latter with the information necessary to update a priority map for saccade guidance. This view was further supported by the finding that higher activity in the right SMG during fixations on the search target was correlated with shorter response times, indicating the right SMG’s involvement in the integration of visual input for object recognition. Taken as a whole, these findings support the hypothesis that the temporoparietal junction (SMG and STG) is a central part in the connectivity networks during guided visual searching and that it is involved in object recognition and the guidance of eye movements.

## Data Availability Statement

The ROI time courses supporting the conclusions of this article will be made available by the authors, without undue reservation.

## Ethics Statement

The studies involving human participants were reviewed and approved by the Ethics Committee of RWTH Aachen University Hospital (EK 225/17). The participants provided their written informed consent to participate in this study.

## Author Contributions

CK developed and designed the methodology, performed the formal analysis, and wrote the original draft of the manuscript. CK and FB set up the experiment and wrote the software for conducting the experiment. JI was involved in the formal analysis, reviewed, and edited the manuscript. RW reviewed and edited the manuscript. NS provided resources for conducting the experiment and reviewed and edited the manuscript. SG and JD conceptualized the experiment, devised the methodology, acquired funding, edited, and reviewed the manuscript. JD was responsible for the project administration and supervision. All authors contributed to the article and approved the submitted version.

## Conflict of Interest

The authors declare that the research was conducted in the absence of any commercial or financial relationships that could be construed as a potential conflict of interest.

## Publisher’s Note

All claims expressed in this article are solely those of the authors and do not necessarily represent those of their affiliated organizations, or those of the publisher, the editors and the reviewers. Any product that may be evaluated in this article, or claim that may be made by its manufacturer, is not guaranteed or endorsed by the publisher.
