# Supplementary material for: Revealing Whole-Brain Causality Networks During Guided Visual Searching
Source: Front Neurosci. 2022 Feb 18;16:826083. doi: 10.3389/fnins.2022.826083 (PMC8894880; doi:10.3389/fnins.2022.826083)
Supplement: Supplementary file 1 [file Data_Sheet_1.docx]

***Supplementary Material: Revealing Whole-Brain Causality Networks During Guided Visual Searching"?***

# Supplementary Figures

## Correlation analysis


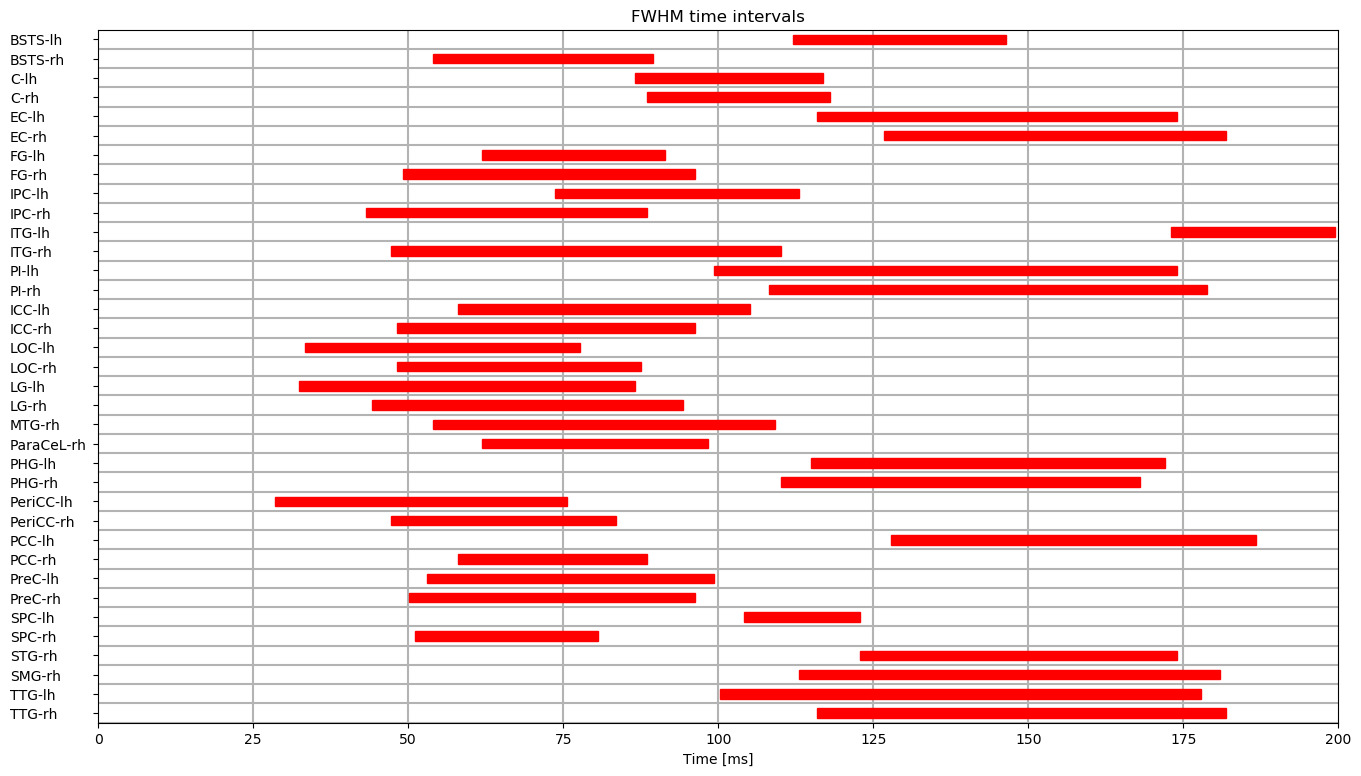


Supplementary Figure 1: The full-width-at-half-maximum (FWHM) time intervals for the grand average ROI FRA time courses.

## Causality

##
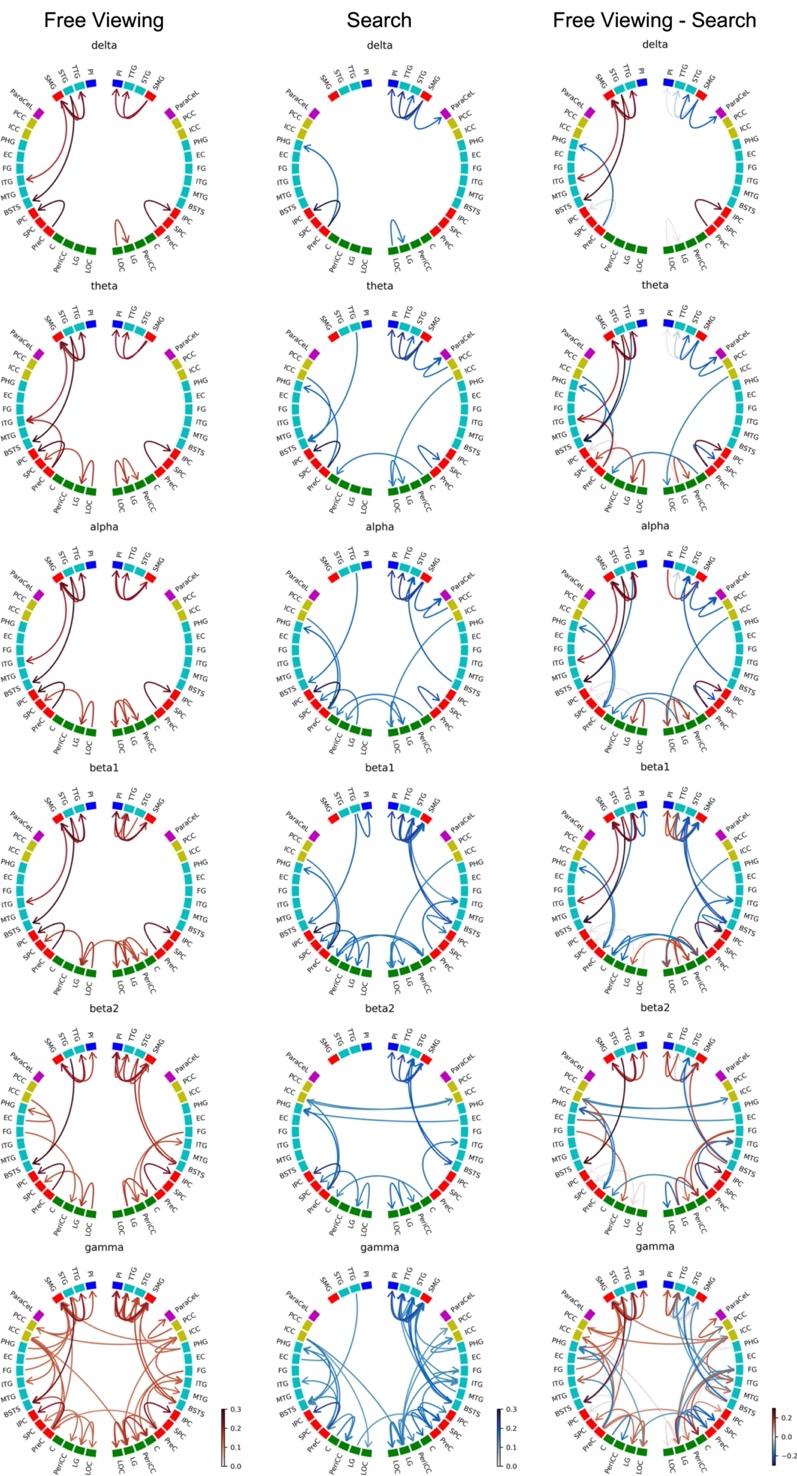


Supplementary Figure 2: Causality group results (GPDC) for fixation onsets during FV, VS, and the direct comparison between FV and VS where the causality matrix containing the group results for VS was subtracted from the causality matrix containing the group results for FV. Red arrows indicate that the connection was stronger during FV, while blue arrows indicate that VS dominated. Grey arrows indicate that the connections were of comparable strength during FV and VS. The ROIs are ordered by hemisphere and cluster (TPC and DC). The color of the nodes indicates the anatomical higher-level region (frontal lobe: magenta, insula: blue, cingulate cortex: yellow, temporal lobe: turquoise, parietal lobe: red, occipital lobe: green).


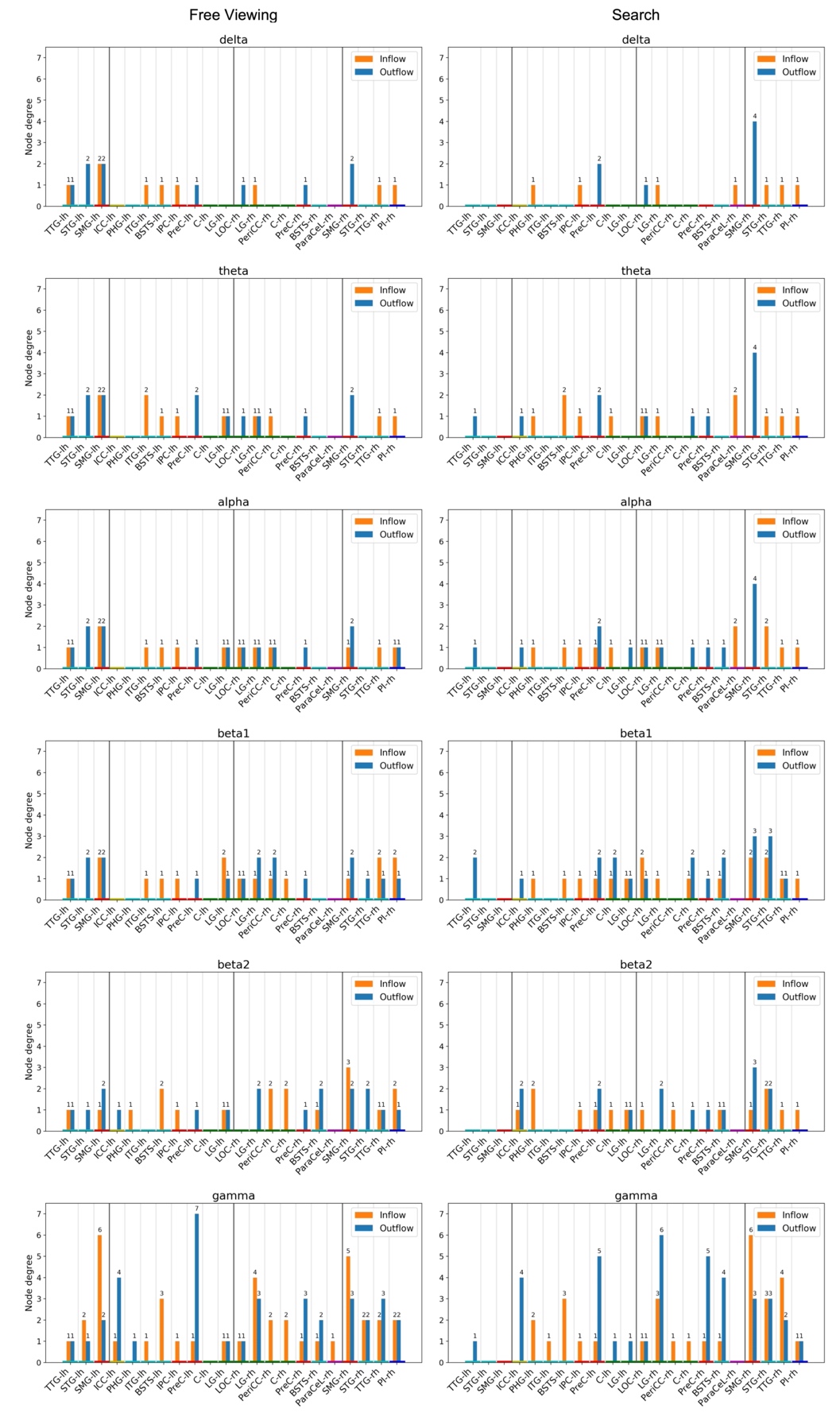


Supplementary Figure 3: Node degree, i.e., the number of incoming and outgoing connections per ROI. Only ROIs with a node degree equal to or above the 95th percentile in at least one frequency band for either task were included. The ROIs are ordered by hemisphere and cluster (TDC and DC). The color of the nodes indicates the anatomical higher-level regions (frontal lobe: magenta, insula: blue, cingulate cortex: yellow, temporal lobe: turquoise, parietal lobe: red, occipital lobe: green).
